# ANASTASIA: An Automated Metagenomic Analysis Pipeline for Novel Enzyme Discovery Exploiting Next Generation Sequencing Data

**DOI:** 10.3389/fgene.2019.00469

**Published:** 2019-05-24

**Authors:** Theodoros Koutsandreas, Efthymios Ladoukakis, Eleftherios Pilalis, Dimitra Zarafeta, Fragiskos N. Kolisis, Georgios Skretas, Aristotelis A. Chatziioannou

**Affiliations:** ^1^Institute of Chemical Biology, Medicinal Chemistry and Biotechnology, National Hellenic Research Foundation, Athens, Greece; ^2^e-NIOS Applications PC, Athens, Greece; ^3^Laboratory of Biotechnology, School of Chemical Engineering, National Technical University of Athens, Athens, Greece

**Keywords:** metagenomics, bioinformatics, next generation sequencing, automated pipelines, systemic biology, novel enzymes

## Abstract

Metagenomic analysis of environmental samples provides deep insight into the enzymatic mixture of the corresponding niches, capable of revealing peptide sequences with novel functional properties exploiting the high performance of next-generation sequencing (NGS) technologies. At the same time due to their ever increasing complexity, there is a compelling need for ever larger computational configurations to ensure proper bioinformatic analysis, and fine annotation. With the aiming to address the challenges of such an endeavor, we have developed a novel web-based application named ANASTASIA (automated nucleotide aminoacid sequences translational plAtform for systemic interpretation and analysis). ANASTASIA provides a rich environment of bioinformatic tools, either publicly available or novel, proprietary algorithms, integrated within numerous automated algorithmic workflows, and which enables versatile data processing tasks for (meta)genomic sequence datasets. ANASTASIA was initially developed in the framework of the European FP7 project HotZyme, whose aim was to perform exhaustive analysis of metagenomes derived from thermal springs around the globe and to discover new enzymes of industrial interest. ANASTASIA has evolved to become a stable and extensible environment for diversified, metagenomic, functional analyses for a range of applications overarching industrial biotechnology to biomedicine, within the frames of the ELIXIR-GR project. As a showcase, we report the successful *in silico* mining of a novel thermostable esterase termed “EstDZ4” from a metagenomic sample collected from a hot spring located in Krisuvik, Iceland.

## Introduction

DNA sequencing techniques have advanced at a prodigious rate during the last decade, attaining higher yields in conjunction with minimizing costs per sequencing run (Stein, 2010). This culminates with the advent of next-generation sequencing (NGS) comprising rapid, high-throughput protocols that generate large amounts of high quality data of deep coverage for only a small fraction of the cost of traditional sequencing technologies (i.e., Sanger). The continuous advancement of NGS techniques has resulted in a subsequent rapid progress in the field of metagenomics, revolutionizing the methodologies for in-depth exploration of the genomic and subsequent enzymatic content of microbial communities without the need of prior culturing. This development, however, is bringing up numerous challenges in the processing of the resulting raw data, thus rendering the bioinformatic analysis a major bottleneck, in any metagenomics project (Scholz et al., 2012). The arising bioinformatic challenges originate from the massive amount of raw data that NGS techniques generate, but also from the vast diversity of bioinformatic tools essential for all gene detection, and annotation tasks that constitute a full metagenomic analysis pipeline (Kunin et al., 2008). In addition, the immense demand on computational and storage resources for such an analysis practically imposes the use of cloud computing methodologies, so as to ensure its feasibility in real world datasets (Wilkening et al., 2009). To address these issues we have developed ANASTASIA (automated nucleotide aminoacid sequences translational plAtform for systemic interpretation and analysis), a user-friendly, web-based, computational infrastructure, which comprises numerous bioinformatic tools, integrated as modular components of automated workflows, and which has been explicitly designed to handle a wide range of metagenomic analyses. This framework is an extended instance of the Galaxy platform (Goecks et al., 2010), customized specifically for metagenomics-related bioinformatic analyses and is linked to a MariaDB database (The MariaDB Foundation, 2009) server via a Web2py (Pierro, 2010) web interface, in order to facilitate the efficient management of the resulting data files. This framework retains all the features of a classic Galaxy instance thus offering the option of being a portable solution while enabling its further customization by integrating additional tools and workflows. ANASTASIA has been tested extensively in diverse metagenomics datasets, as it was initially developed to be the core bioinformatics tool of the EU FP7 project HotZyme^1^, which targeted to the exhaustive analysis of metagenomes from thermal springs (Wohlgemuth et al., 2018), with the scope of evolving into a stable environment for powerful, and bionformatic analyses that may support a broad range of metagenomics analysis tasks. ANASTASIA is certainly the first of its kind platform, with this level of operational maturity and tested stability, to provide all-inclusive workflows, handling each part of the long, and diverse list of steps comprising an exhaustive analysis of metagenomic data, being at the same time, fully customizable by its users. In contrast to the limited analytic options and limited automation offered by other pipelines with the same broader scope, ANASTASIA’s automated pipelines are able to process various computational steps, from handling raw sequencing data to assigning putative function predictions for gene encoding sequences, or providing powerful functional characterization of the underlying emerging molecular networks. Based on all the aforementioned, it is capable of addressing different problems, like the screening of thermophiles or the systematic screening of the human microbiome in various infections, as part of the Hellenic Bioinformatic computational Infrastructure ELIXIR-GR, which represents the Hellenic node of ELIXIR.

## Materials and Methods

### Design and Implementation

The backbone of the ANASTASIA platform was assigned to a server by the name “Motherbox” owned by the National Technical University of Athens (NTUA) that hosted a local instance of the Galaxy platform. The Motherbox server is equipped with 64 CPU cores, 512 GB RAM and a total of 7.2 TB disk capacity. The Galaxy installation was performed by downloading the latest version of the source code from the Mercurial (Mercurial, 2005) depository^2^ and running its startup script (run.sh) in order to automatically download all dependent Python (The Python Programming Language, 2001) modules (“eggs”) that are essential. As part of the installation process, the local MariaDB database server was exploited and installed on Motherbox and linked to the Galaxy server through integration of the appropriate custom initialization arguments in the system configuration file (galaxy.ini) of the platform. The web accessibility of Galaxy was secured by configuring Motherbox’s Apache web server (The Apache, 1995) to proxy any requests to the virtual host motherbox.chemeng.ntua.gr/anastasia_dev/ to a dedicated port on the server, thus broadcasting the platform to all external users via the aforementioned web address. Extra customization to the configuration files of the Galaxy instantiation to include and specify the bioinformatic tools and algorithms available after integration in order to become embedded in ANASTASIA’s front-end list of tools.

### Front-End Customization

The ANASTASIA front-end was developed by altering the graphical user interface (GUI) of the Galaxy platform into a more intuitive and user-friendly layout. The new GUI, was based on scripts written in JavaScript (Javascript, 2016) that modify the typical settings of the homepage layout of the Galaxy platform and that add several different menu and submenu buttons, which correspond to distinct categories of the integrated bioinformatic tools and available workflows. Most of the tools that were incorporated into the original Galaxy instance were removed and have been replaced by appropriately curated and tested tools designed for metagenomic analytical tasks, such as *de novo* sequence assembly, open reading frame (ORF) detection, homology based protein prediction, machine-learning-based protein prediction, etc. This was accomplished by developing tuned parser algorithm scripts in Perl (The Perl Programming Language, 2002) and Python language, as well as by preparing appropriate configuration files in Extensible Markup Language (XML) (Extensible Markup Language, 2013) for each individual tool. In that way, the integration of each tool was managed by means of a Galaxy-generated GUI (written in XML) that links the necessary input parameters into the parser script which, in turn, invokes the corresponding executable. The choice of tools that were installed on the server and integrated into ANASTASIA was based mainly on three criteria: (1) their overall performance vs. their computational cost plus resource demands, (2) their compatibility with the Galaxy platform, and (3) their user-friendliness (i.e., advanced visualization functionality, interactive data management in local computers, such as MEGAN). While the first criterion is important for tasks, such as contig assembly and sequence similarity searches, which constitute the computational bottlenecks of every bioinformatic analysis of sequencing data, the latter two were the main consideration for less costly parts of the analysis, but of great importance regarding the quality of the interpretation. In addition to already established bioinformatic tools, the novel interface of ANASTASIA was enriched with in-house algorithms that enable numerous data processing tasks such as functional analysis and data management among others (see below).

### Data Acquisition

A tool for direct file uploading to the platform was included in the original Galaxy instance and was kept available as it enables both access to local data from a user’s personal computer, as well as data available online via URL. In addition, numerous sequencing raw data files were linked to ANASTASIA via their directory path in the Motherbox server e.g., large sequencing datasets from the HotZyme project. This enables their direct utilization by each user precluding the creation of additional copies for each analysis. This became feasible by altering Galaxy’s system configuration files and may be used for any dataset import on the server, provided the availability of its directory path.

### Sequencing Quality Control

Sequencing quality is expressed based on Phred base calling algorithm (Ewing et al., 1998), which assigns a quality score Q to each base, proportional to its error probability P. Each score is calculated using the formula: Q = -10log_10_P. The data from any sequencing experiment generate a second file containing the scores matching the sequencing reads from the first one or, more commonly, combine the two in the same file, i.e., FASTQ format. Any sequencing quality control analysis requires a tool to parse through the sequencing data files and according to the user’s specifications either trim or exclude highly problematic reads. For such a task there are various bioinformatic solutions (Davis et al., 1998; FastQC, 2010; Fastx Toolkit, 2009; Pandey et al., 2016), all of which manage to handle the issue quite rapidly, and memory efficiently (Davis et al., 1998). In order to enable sequencing quality control in ANASTASIA and considering the somewhat similar performance of all the above-mentioned tools, the FASTX toolkit (Fastx Toolkit, 2009) and FastQC (FastQC, 2010) were chosen for integration as they were already included in the original Galaxy instance. These tools were installed on Motherbox server and their corresponding Galaxy parsers and XML configuration files were preserved in the final platform.

### Taxonomic Analysis

There are two major identification schemes for microbial species in a metagenomic sample, the first being based on amplicon sequencing (e.g., 16S rRNA), while the latter on whole metagenome sequencing (Garrity, 2016). Since this first version of ANASTASIA is currently dedicated to analyzing whole metagenome sequencing datasets and as this approach for taxonomic classification has been proven more efficient than amplicon sequencing (Escobar-Zepeda et al., 2018), the appropriate tools had to be considered for integration. The MEGAN (Huson et al., 2007) software was chosen, due to its advanced operational features, including both Linux and Windows support, providing an interactive GUI for further result visualization and interactive data management on a personal computer, as well as its potential to be used as a metabolic pathway analysis tool, exploiting reference databases such as KEGG (Kanehisa et al., 2017), SEED (Overbeek et al., 2005), and COG (Tatusov et al., 2000). The tool operates by receiving the resulting dataset from a sequence similarity search analysis such BLAST as input, in order to calculate the percentage of each species present in the sample while yielding summarized results as visual representations (e.g., bar charts).

### *De novo* Sequence Assembly

One of the major bottlenecks, in terms of execution times and computational demands, is the assembly of reads into contiguous sequences (contigs), mostly due to the ever higher throughputs of NGS technologies, of small read lengths. Consequently, the sequence assembly methodologies transition from the overlap-layout-consensus (OLC) algorithms toward De-Bruijn-graph based paradigms (Li et al., 2012). The choice of the proper assembler relies on two equally important points: (1) the quality of the produced assembly and (2) the required computational resources. Numerous studies have managed to extensively compare various assemblers either for metagenomic data (van der Walt et al., 2017; Vollmers et al., 2017) or for single organism genomic data (Earl et al., 2011; Bradnam et al., 2013) and while there seems to be a slight advantage of metaSPAdes (Nurk et al., 2017), in terms of length of the produced contigs, the final choice for ANASTASIA was Megahit (Li et al., 2015) as it attained a comparable performance with much smaller computational resources than the typical configuration of metaSPAdes. The Velvet (Zerbino and Birney, 2008) assembler was also included in the final instance because of its popularity, as it provides visualization options for its assembly via the generation of afg files and is already included in the repository for Galaxy tool integration Galaxy Tool Shed^3^. Both tools were installed on the Motherbox server and later linked to the platform via the appropriate changes in Galaxy’s tool configuration file (tool_conf.xml). Megahit was configured manually via an in-house XML script integrating it to ANASTASIA.

### ORF/Gene Detection

*De novo* gene detection, while not being significantly challenging in terms of computational cost, is the most crucial part in any analysis oriented toward unearthing novel enzymes from metagenomic samples. From an algorithmic perspective, detecting a gene in a large contiguous sequence could theoretically require only the extraction of the appropriate ORF as performed by the Getorf tool of the EMBOSS suite (Rice et al., 2000). Nevertheless the existence of an ORF cannot guarantee that the specific genomic region is translated as is. For example, spurious ORFs (Veloso et al., 2005), especially in high GC content genomes, can often lead to the detection of numerous false potential gene sequences within the same region. Moreover, sequence assembly can sometimes be omitted entirely or, depending on parameter settings, read coverage and abundance of species, result in shorter contigs that correspond to incomplete genomes thus rendering potential ORFs undetectable. Various algorithms (Noguchi et al., 2006, 2008; Rho et al., 2010; Zhu et al., 2010) have emerged that are dedicated to working with short sequences (either shorter contigs or even reads) but as an extensive third-party benchmarking study is yet to be published, FragGeneScan tool was the choice for integration with ANASTASIA as it seems to outperform all its predecessors (Rho et al., 2010). This tool was installed on the Motherbox server but its wrapper script (run_FragGeneScan.pl) had to be edited, in the way it produced the output files, in order to be more compatible with a Galaxy instance that would host numerous users simultaneously. The approriate XML configuration files for integration with ANASTASIA were imported by Galaxy Tool Shed and edited appropriately in order to comply with the abovementioned changes in the wrapper script.

### Protein Function Prediction

In order to predict the putative function of the sequences retrieved from the ORF/gene detection analysis of the BLAST suite (Altschul et al., 1990) alignment tools and the HMMER (Finn et al., 2011) software were installed on the server and integrated on ANASTASIA by using the publicly available XML configuration files found in Galaxy’s Tool Shed (Galaxy Tool Shed, 2005). For the BLASTn and BLASTp tools, which have been designed to handle nucleotide and amino-acid sequences respectively, the NCBI-nt, NCBI-nr and UniProt databases were downloaded on the server and were parsed with the appropriate BLAST commands (makeblastdb) for immediate use. Moreover, for the purposes of HotZyme project, code was written in Perl that enabled the creation and formatting of a BLAST-oriented, customizable database, which contained all of the annotated sequences with hydrolytic activities derived from the SwissProt (Apweiler et al., 2004) database. For the HMMER tool, the Pfam (Sonnhammer et al., 1997) database was downloaded and formatted for use with the appropriate commands (hmmpress). To enrich ANASTASIA’s protein prediction capabilities, we applied machine-learning based methodologies that enable us to translate the genomic content of any environmental sample. These include the installation and integration into ANASTASIA of the EFICAz (Kumar and Skolnick, 2012) software and of the PROKKA (Seemann, 2014) pipeline, which, in spite of the numerous programs, it calls upon to perform a complete analysis, is introduced to the user as a single tool.

### Data Management/ANASTASIA Knowledgebase Design

To avoid transactional locks generated with the increase of the platform’s users and to improve overall efficiency, ANASTASIA was linked to a local MariaDB database on Motherbox by editing the Galaxy setup configuration file (galaxy.ini). In order to facilitate the parsing of resulting data (e.g., contig assembly or BLAST result files), in-house algorithms written in Python were integrated as tools in the platform. Each Python parser imports the corresponding data into the MariaDB database on the Motherbox server and returns it as a dump file allowing the user to download it and re-import it into their local MariaDB or MySQL (MySQL, 1995) database server. Additionally, the parsers designed to handle BLAST results, return FASTA files with all the sequences from the original dataset that do not return any alignment hit above the predetermined statistical thresholds. For the purposes of online inspection and user interaction with the scope of filtering analysis results, the ANASTASIA Knowledgebase was developed, a MariaDB database system that allows users to import their results (as MariaDB/MySQL dump files) and access them via a Web2py interface. This was implemented through an in-house tool developed in Python and integrated into ANASTASIA. This tool creates a new database schema, where it imports the data and appends the new schema information in Web2py’s “controller” Python scripts. In this way the schema is afforded via a user interface, which also contains all MariaDB query search capabilities for quick and efficient data manipulation, filtering, and retrieval. The created web interface is secured via an access control system, which the user has to apply for in order to get the necessary username and password credentials from an administrator. The tool generates a unique id for each database entry, available only to the user, with which the data can be retrieved at any later time. Each user can access the knowledgebase either directly from ANASTASIA’s front end or from the link generated from the tool that includes also the unique id for their datasets.

### Supplementary Tools

Additional tools, based on both published and in-house scripts, have also been integrated in the platform and perform various non-trivial tasks such as sequence clustering (Li and Godzik, 2006), sequence translation (Fastx Toolkit, 2009), and data format management, etc. These tasks have also been incorporated in the subsequent workflows (see *Metagenomic workflows)* in order to format datasets accordingly for optimal utilization in each different part of the analysis by the respective tool(s).

### Metagenomic Workflows

The aforementioned tools have been assembled together as modules of automated workflows in ANASTASIA that can handle any type of metagenomic dataset and analysis. These workflows enable their comprised tools to exchange input and output data with each other, in order for each one of them to execute the specific analytical task, automatically, without any user interference e.g., the gene detection tool will automatically use the output data from the assembly tool and will in turn provide its output data to the BLAST tool for further annotation. This was made possible by exploiting Galaxy’s Workflow Canvas, in order to design the sequel of the various analytical processes, or by simply extracting the history of each of our past analysis in the HotZyme project as a complete, and ready to use workflow. These capabilities of the Galaxy application were retained in ANASTASIA so as to inherit the users with the capability to create their own customized workflows on top of the default ones we include. ANASTASIA comes with default automated workflows that allow a complete analysis depending on the input data and are named accordingly. In addition, these workflows might also be fully customizable if imported to a user’s account, something which may prove extremely useful and extend their functionality. This can be exploited for instances on machines with limited resources where handling computationally intensive parts of the analysis, e.g., similarity searches via BLAST is essential. The “Starting From FASTQ Reads” workflow is designed for raw sequencing reads in FASTQ format as input and includes the following tasks: (i) quality control by FASTX toolkit; (ii) assembly into contigs by Megahit; (iii) gene identification by FragGeneScan; (iv) gene annotation using a combination of BLAST, PROKKA, HMMER and EFICAZ; (v) visualization of results using in-house parser scripts that import the annotation results into the server’s MariaDB database and visualize it via Web2py. The “Starting From FASTA Reads” workflow has the same functionality as the one mentioned above but is designed for FASTA formatted datasets hence omitting the quality control steps. The “After Assembly” workflow follows all the previous annotation steps but starting with contig FASTA files as input i.e., the results of the assembly process. The “Taxonomic and functional analysis” workflow is designed for detecting the different microbial populations in a metagenomic sample and requires as input a FASTA file of raw sequencing reads that subjects to the following analysis: (i) homology analysis using BLAST tool against NCBI-nr database, (ii) taxonomic analysis using MEGAN software. Every workflow consists of tools and algorithms integrated in ANASTASIA with each and every one of them being available as a standalone tool for the user to exploit in customized analyses.

### Biotranslator Workflow

Functional enrichment analysis constitutes the foremost approach to interpret the impact of a set of genes (or gene products) to the cellular physiology, namely the co-regulation of distinct cellular mechanisms that gives rise to diverse phenotypes. Conceptually, it is based on the association of genes with semantic terms, which refer to molecular pathways, cellular components, biological mechanisms, or phenotypic traits. Those terms are predominantly organized in logical structures, which describe the knowledge of a specific biological domain (Gross et al., 2016). In order to aid the elucidation of the biological underpinnings of an unknown gene set, each term needs to be annotated with genes, and based on prior inferences of scientific studies. Gene Ontology (Ashburner et al., 2000), KEGG (Kanehisa et al., 2017), and reactome pathways (Fabregat et al., 2018) are well-established omnibuses, which correlate systematically their descriptive terms with genes by following a hierarchical structure of deductive steps and constructing the appropriate framework for the functional enrichment analysis. However, as the scientific community is mainly focused on organisms of traditional biomedical interest, they produce electronically, and manually curated annotations only for a limited organismal spectrum, including human, mouse, other model organisms and some specific bacteria, which are a negligible ratio of the whole prokaryotes’ kingdom.

Nowadays, various tools and software are used for the functional enrichment analysis of significant gene lists, derived from -omics experiments. Their core computational process consists of over representation of statistical tests and *p*-value correction approaches, in order to minimize the amount of false positives. The StRAnGER algorithm (Chatziioannou and Moulos, 2011) applies a non-parametric procedure, targeting to mitigate experimental and annotation noise by filtering out any trivial and non-informative term and reveal system-level terms, which reflect the underlying components of the examined phenotype. As a result, it translates the individual genes, through the aforementioned vocabularies, to a restricted list of prioritized terms, which could be depicted as a broad network of functional of phenotypic entities.

This network represents a descriptive snapshot of the biological problem under investigation, incapable to condense the interpretation to few precise markers. GOrevenge (Moutselos et al., 2011) has been developed to surpass that limitation of functional enrichment analysis, aiming to propose potential regulatory hub genes or gene products, or even further putative marker signatures, related to the ranked set of over-represented terms. It uses graph-theoretical methods, exploiting the graphical structure of the controlled databases, such as the direct acyclic graph of GO, to detect cross-linked entities, which take part in many topologically distinct nodes of the graph. In this way, the output of an omic experiment may result to a compact list of prioritized genes or proteins, without the use of prior knowledge, or human supervision (i.e., phenotype related seed sets or keywords), reducing problem dimensionality to a succinct set of features.

As our motivation here was to provide automated solutions for the functional description of microbial communities, the aforementioned approaches sought adaptive adjustments. A common processing of metagenomic raw data correlates the detected ORFs with known sequences or protein domains, based on sequence alignment algorithms. The functional interpretation could rely on those sequence similarity predictions. Besides the debatable assumption that sequence similarity implies or not functional relevance (Gerlt and Babbitt, 2000), the overriding problem is that the existed functional enrichment analysis tools use organism-specific data, in contrast to the fundamental concept of metagenomics studies. Metagenomics explore the microbial communities as a unified entity, endeavoring to detect and decipher the synergistic mechanisms that cause community homeostasis or other biotechnologically interesting features (such as thermostability and resilience to acid environments). As a result, an appropriate functional enrichment analysis tool needs to take into account all the available functional annotation of the prokaryotic world, combined in a unified database.

UniProt-SwissProt knowledgebase (Apweiler et al., 2004) includes manually curated descriptions of more than 350k known proteins from the prokaryotic world. To overcome the existence of organism-specific databases, such as *Escherichia coli* or Bacillus subtilis, we exploited the whole mapping of UniProt-SwissProt knowledgebase. We combined data from different organisms (bacteria and archaea), related to gene products and GO terms associations and producing a unified schema for GO. All amino acid sequences, which originated from the same gene in different organisms, were conceptually clustered together, combining their functional annotations to produce a unified gene – GO terms mapping. In order to eliminate the annotation bias of extensively studied prokaryotes, infrequent associations were filtered out. The relative frequency of each gene – GO term pair was calculated as the ratio of organisms which include that pair in their mapping to all the organisms, which contain that gene in their DNA. Gene-specific distributions of relative frequencies were constructed so that every pair with value lower than the respective distribution median was excluded from the final annotation schema. The output graphs of GO constitute a global description of biological processes, cellular components and molecular functions that exist in the prokaryotic kingdom and are correlated with at least one gene, independently of taxonomic details. Such an ensemble of annotations could be used for the biological interpretation of microbial communities, regardless their population distribution, and taxonomic profile.

Summarizing, in the framework of the ANASTASIA platform, a new workflow was developed, named BioTranslator, for the functional interpretation of metagenomic data, which encompasses sequential computational steps, and adapted to the particularities of input data. In order to analyze a metagenomic sample, the user is able to import either a BLASTp output (specific tabular format and executed on the SwissProt database) or a list of gene symbols, derived from previous analytical tasks. Targeting the detection of the most trustworthy BLAST hits, BioTranslator adopts strict alignment criteria, filtering out matches with query coverage lower than 90% or subject coverage lower than 50% while taking into consideration a user-defined threshold about the hits’ *e*-value. The best hit of each query is kept and UniProt IDs are translated into the respective gene symbols. Regardless of the initial input, StRAnGER performs the functional enrichment analysis of genes list and derives a set of statistically significant terms, as they are described in the three GO domains. The user defines the domain which will be used for the prioritization of genes. Hence, GOrevenge uses the enriched part of that domain in order to exploit its topological characteristics and disclose the most critical genes that could be assumed as the master regulators, bearing a part of causality of community features.

### Application of ANASTASIA

Automated nucleotide aminoacid sequences translational plAtform for systemic interpretation and analysis was exhaustively tested in various aspects, during the HotZyme project where it was mainly used to store, manage and annotate metagenomic sequencing data, taken from eight remote hot springs around the world (Menzel et al., 2015), in order to detect novel thermostable enzymes of industrial interest. The first beta version of the platform was installed on a server of the University of Copenhagen named “Helios” and provided access both to the data and to the annotation tools in order to predict sequences that correspond to thermostable enzymes of potential hydrolytic activity which could be verified in the lab at a later point. In this first version, during the development of its various automated workflows and corresponding modules, we exploited each integrated algorithm to run our first analyses of the samples, which, in turn, resulted in the detection of various novel enzymes exhibiting enhanced thermostability (Zarafeta et al., 2016a,b). The final version of ANASTASIA was fed again with pre-existing data for another iteration of the analysis but this time via its automated workflows of fined-tuned algorithms (supplied with default parameters for optimal performance) and resulted in the detection of an additional novel enzyme, termed EstDZ4, as described below.

### Identification of the estDZ4 Gene

The raw sequencing data from each sample of the HotZyme project were imported in the server Helios of the University of Copenhagen and were linked to ANASTASIA, thus making it directly available both for download and analysis via its integrated tools and workflows. Sequencing assembly was performed (Menzel et al., 2015) from University of Copenhagen and resulting contig datasets were also imported in ANASTASIA for further analysis. The selection of the sequence of *estDZ4* as a candidate gene encoding a protein with putative esterolytic activity occurred through the application of the “After assembly” workflow on the assembly data of the sample Is3-13 originating from a high temperature pool (90°C/pH 3.5–4.0) in Krisuvik, Iceland (Menzel et al., 2015). The assembly dataset of the Is3-13 sample was 29.8MB in size and consisted of 34.651 contigs originating from 10,050,000 raw reads. Running time on the Motherbox server (512GB RAM, 64CPUs) for the same workflow, using the above-mentioned dataset, lasted 24 h, 54 min, and 20 s with the major bottlenecks being, as expected, the similarity searches to the locally downloaded databases of NCBI-nr (15 h, 27 min, and 50 s) and Pfam-A (7 h, 27 min, and 34 s). The results were downloaded as sqldump files and examined on MySQL workbench where the sequence for EstDZ4 was chosen for further curation. EstDZ4 was chosen because it exhibited 99% identity (query coverage 98%) to a putative esterase/lipase from *Thiomonas sp.CB3* [GenBank: CQR41430.1] in NCBI-nr but only 23% identity (query coverage 84%) to a previously characterized GDSL esterase/lipase from *Arabidopsis thaliana* [UniProtKB/Swiss-Prot: Q9FIA1.1]. Futhermore, it was predicted to contain a GDSL-like Lipase/Acylhydrolase catalytic domain from Pfam-A database.

The representative (non redundant) putative gene sequences detected from the initial steps [FragGeneScan and CD-HIT (Li and Godzik, 2006)] of the above mentioned pipeline were used as input in the BioTranslator workflow, producing the pathway analysis results in a total of 36 min and 5 s. Once again, the similarity search analysis (BLASTp against a customized database containing only the prokaryote entries from SwissProt) was the major bottleneck requiring a total 31 min and 37 s.

### Cloning, Purification, and Biochemical Characterization of EstDZ4

Construction of the pASK-EstDZ4 plasmid was carried out by amplifying *estDZ4* from the isolated metagenomic DNA by polymerase chain reaction (PCR) using primers containing an XbaI site (5′- AAAAATCTAGAAGGAGGAAACGATGTCCGTGGCGAGTGTGAATTCGGCC-3′) and an XhoI site along with octahistidine tag (5′- AAAAAACTCGAGTTAGTGGTGGTGGTGGTGGTGGTGGTGTTGCGAAATCCAGCCAAAACCC-3′) (restriction sites underlined, octahistidine tag doubly underlined). The forward primer was designed so as not to include amino acids 1–38, which were predicted to correspond to a signal sequence according to HMMER analysis. The PCR product was cloned into the expression vector pASK75 (Skerra, 1994). *E. coli* Origami 2 (DE3) (Novagen) cells were transformed with pASK-EstDZ4, grown in 5 ml of Luria-Bertani (LB) medium containing 100 μg/ml ampicillin at 37°C with shaking until the culture reached an optical density at 600 nm (OD600) of 0.5, at which point 0.2 μg/mL anhydrotetracycline (aTc) were added to induce *estDZ4* overexpression. The cells were collected, lysed by brief sonication and the clarified lysates were used to run a zymogram as described before (Zarafeta et al., 2016b). Briefly, the gel was rinsed in distilled water and incubated for 30 min at 37°C in 0.1% Fast Red TR-salt (4-chloro-2-methylbenzenediazonium salt) in 0.1 M Tris–HCl buffer (pH 7.0) containing 2% of a 1% (v/v) 1-naphthyl acetate solution in acetone. Esterolytic activity was visualized by the appearance of a band ofred-brown color.

For EstDZ4 purification, Origami 2 (DE3) cells carrying the pASK-EstDZ4 plasmid were grown as described above, and the induction of *estDZ4* overexpression was performed by overnight incubation at 25°C with shaking. The cells from a 500 mL culture grown in a 2 L shake flask were harvested, washed, re-suspended in 10 mL equilibration buffer enhanced with 1% Triton X-100 (v/v) and lysed by brief sonication steps on ice. The cell extract was clarified by centrifugation at 10,000 × *g* for 15 min at 4°C and the supernatant was collected and mixed with 0.5 mL Ni-NTA agarose beads (Qiagen – Hilden, Germany) and agitated mildly for 1 h at 4°C. The mixture was then loaded onto a 5 mL polypropylene column (Thermo Fisher Scientific – Waltham, United States), the flow-through was discarded, and the column was washed with 10 mL of NPI20/ Triton wash buffer followed by a second wash with non-Triton-enriched NPI20. EstDZ4 was eluted using 1 mL of NPI200 elution buffer. All buffers used for purification were prepared according to the manufacturer’s protocol (Qiagen – Hilden, Germany) unless stated otherwise. EstDZ4 was further purified by size-exclusion chromatography (SEC) using a Superdex75 10/300GL column (GE Healthcare, United States).

Protein concentration was estimated according to the assay described by Bradford (Bradford, 1976) using bovine serum albumin as a standard. The purified protein was visualized by SDS-PAGE analysis and staining with Coomassie blue or western blotting using an anti-polyhistidine monoclonal antibody conjugated with horseradish peroxidase (Sigma – St. Louis, MO, United States).

The catalytic activity of EstDZ4 was determined by quantification of the amount of *p*-nitrophenol (pNP) released from pNP ester substrates by photometric measurement at 410 nm. Unless stated otherwise, the 100 μL standard reaction mixture consisted of 25 mM phosphate buffer pH 6.5 enriched with 0.05% Triton X-100 (v/v), 2 mM pNP-octanoate and 1 μg/mL of pure enzyme, and was carried out for 5 min at 75°C on a MJ Research thermal cycler, with a pre-incubation setting of the buffer to the target temperature, before the enzyme was added. Enzymic activity was recorded using a Safire II-Basic plate reader (Tecan, Austria) by measuring the absorbance of the released pNP at 410 nm, immediately after the reaction was completed. For the substrate specificity experiments, a range of different pNP-fatty acyl esters, such as acetate (C2), butyrate (C4), octanoate (C8), decanoate (C10), laurate (C12), and palmitate (C16) were used in the standard reaction. For the determination of the enzyme’s optimal pH, reactions were carried out at 40°C in 25 mM acetate, phosphate, Tris–HCl and glycine-NaOH buffers for pH values 4–6, 6–7, 7–9, and 9–10, respectively. Activity was measured by recording absorbance at 348 nm, the isosbestic point of pNP, so as to exclude the pH effect on the readings. Temperature profiling of EstDZ4 was performed by incubating the standard reaction at temperatures ranging from 25 to 70°C. Thermostability experiments were conducted by incubating the enzyme at high temperatures for prolonged time periods and subsequently measuring its activity in the standard reaction.

## Results and Discussion

### Development of ANASTASIA and Stand-Alone Portability

**Figure 1:**
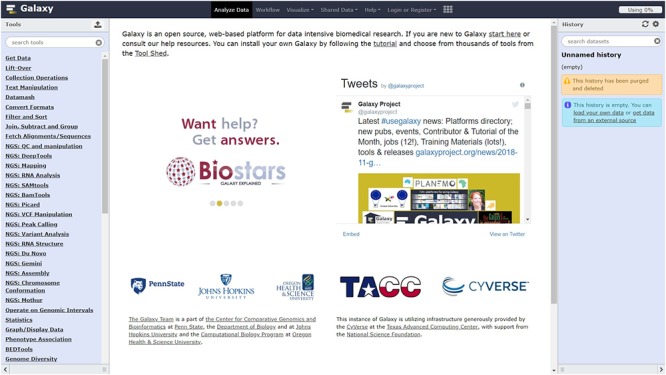
The original Galaxy front end.

**Figure 2:**
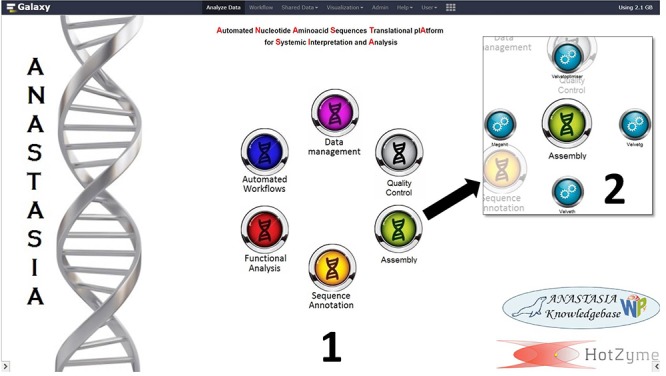
The customized front page of ANASTASIA framework with (1) the main menu buttons and (2) the submenu of “Assembly” suite of tools. The submenu consists of 4 tools that handle NGS assembly from reads to contigs and a tool that imports the output assembly data into a MariaDB database.

**Figure 3:**
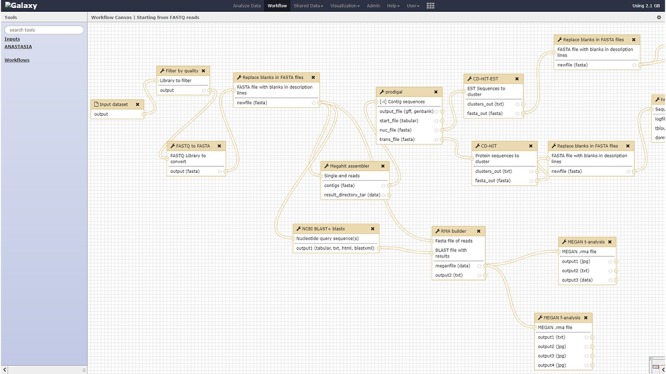
Galaxy’s Workflow Canvas.

**Figure 4:**
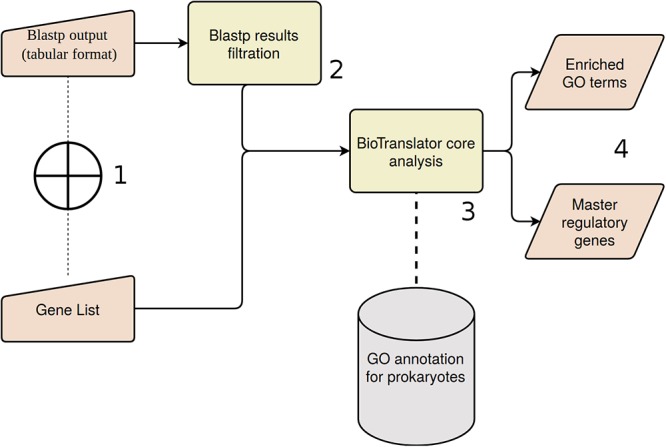
BioTranslator workflow. (1) User could start the analysis with a pre-defined gene list, or sequence similarities derived from Blastp analysis. (2) Blastp results are filtered if the initial input is the respective tabular file. (3) Pathway analysis and master regulators detection are based on BioTranslator core algorithms. (4) Final outputs of BioTranslator analysis.

Automated nucleotide aminoacid sequences translational plAtform for systemic interpretation and analysis was developed as a user-friendly, web-based, computational pipeline, aiming to address numerous and diversified series of metagenomic analysis tasks for the massive characterization of a broad constellation of bacterial metagenomes. For this scope it integrates numerous bioinformatic tools (Figure 1, 2), as components of automated workflows (Figure 3) designed to handle a wide range of processing tasks. In contrast to the limited versatility and automation capacity of other metagenomic pipelines, ANASTASIA’s automated workflows are tackling various processing steps, from the handling of raw sequencing data to the putative function predictions for gene encoding sequences and the powerful functional characterization of the underlying emerging molecular networks (Figure 4). ANASTASIA can address different scenarios, from the screening of thermophiles to the systematic screening of the human microbiome in various infections, as part of the Hellenic Bioinformatic computational Infrastructure ELIXIR-GR, which represents the Hellenic node of ELIXIR. Below, as a showcase of the practical utility of ANASTASIA as an efficient platform for data-driven discovery, we report the successful *in silico* mining of a novel thermostable esterase, termed EstDZ4, as a result of the exhaustive analysis of a metagenomic sample taken from a hot spring located in Krisuvik, Iceland.

Access to ANASTASIA and its tools is possible either via the publicly available server^4^ or as a local bundle installation after download by executing a Bash script^5^. The script, which works in any Linux platform, performs the following actions: (i) automatically downloads the most recent (without compatibility issues) Galaxy instance; (ii) assigns a MariaDB database for this instance; (iii) builds the ANASTASIA interface and configures the web server for online viewing of the platform; (iv) downloads the customized tools and integrates them in ANASTASIA; (v) downloads any essential databases needed for the tools (NCBI-nr, nt, UniProt, etc.) to operate and formats them accordingly to be usable (e.g., makeblastdb tool for creating BLAST-able databases) and (vi) runs the Galaxy startup script (sh run.sh) in order for ANASTASIA to activate.

### Discovery and Biochemical Characterization of EstDZ4

Automated nucleotide aminoacid sequences translational plAtform for systemic interpretation and analysis was used to analyze the metagenomic DNA of the Is3-13 sample originating from a high temperature pool (90°C, pH 3.5–4.0) in Krisuvik, Iceland (Menzel et al., 2015) described above. From this analysis, a specific sequence was selected for further investigation as a proof of concept for the ability of the platform to identify new enzymes. The selected sequence, named *EstDZ4*, was predicted to encode a 454-amino acid protein with a predicted molecular mass of 46.3 kDa. According to a BLAST analysis against the SwissProt/Uniprot database containing characterized proteins, EstDZ4 presented 23% identity (query coverage 84%) to a previously characterized GDSL esterase/lipase from *A. thaliana* [UniProtKB/Swiss-Prot: Q9FIA1.1] (Cheng et al., 2017) and a 99% identity (query coverage 98%) to a putative lipase/esterase from *Thiomonas sp.CB3* [GenBank: CQR41430.1]. The same analysis assigned the protein to the Triacylglycerol lipase-like subfamily of the SGNH hydrolases [NCBI Conserved Domains Datatabase accession number: cl01053 (Marchler-Bauer et al., 2017)], which is a diverse family of lipases and esterases. Sequence analysis against the Pfam-A database using HMMER predicted that the sequence contains a GDSL-like Lipase/Acylhydrolase catalytic domain spanning amino acids 61-442, as well as a signal peptide (amino acids 1-38). Further examination with EFICAz assigned a putative esterolytic activity to the sequence, according to its EC number prediction (3.1.1.).

**Figure 5:**
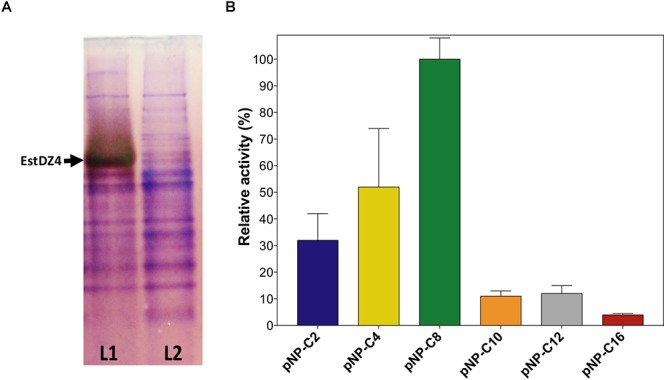
Confirmation of EstDZ4 esterolytic activity. **(A)** Detection of EstDZ4 esterolytic activity via native PAGE analysis and Fast Red staining using 1-naphthyl acetate as a substrate. L1, clarified lysate of cells overexpressing estDZ4; L2, clarified lysate of cells carrying an empty vector. **(B)** Substrate profiling of the esterolytic activity of EstDZ4, using purified enzyme. The relative enzymic activity was measured as released pNP at 410 nm (pH 7, 40°C). The reported values correspond to the mean value of three independent experiments performed in triplicate and the error bars to one standard deviation from the mean value. Assaying the esterolytic activity of EstDZ4 within the pH range of 4–10 at 40°C using pNP-octanoate as the substrate, revealed that the optimal pH for the new enzyme is pH 6.5 (Figure 6A). Measurements of its relative catalytic activity at different temperatures, on the other hand, showed that EstDZ4 has a broad temperature range of action as it retains high levels of esterolytic activity at temperatures between 40–85°C, with its optimal temperature of action being at 75°C (Figure 6B). In order to evaluate the thermostability of EstDZ4, the enzyme was incubated for prolonged time periods in high temperatures and its residual activity was measured. As shown in Figure 6C, EstDZ4 exhibited a half-life of ∼5 h when exposed to 80°C, and even after 24 h of incubation at 70 and 75°C, the enzyme retained more than 40% of its initial activity, demonstrating that EstDZ4 is a highly thermostable esterase.

The *estDZ4* gene was amplified by PCR from the Is3-13metagenomic DNA sample and was cloned (after excluding the predicted signal peptide) into the plasmid pASK75 (Skerra, 1994) to form the vector pASK-EstDZ4. *E. coli* Origami 2 (DE3) cells were transformed with pASK-EstDZ4 and the recombinant protein was produced as described in the Section “Materials and Methods.” To examine the putative esterolytic activity of EstDZ4, a zymogram analysis was performed using clarified lysates of cells producing the recombinant protein and cells carrying an empty vector. Following the separation of the proteins contained in the clarified cell lysates by native PAGE, the gel was exposed to 1-naphtyl acetate as a potential substrate for ester hydrolysis and stained with Fast Red. Immediately upon staining, a band of red-brown color appeared only for the EstDZ4-producing sample (Figure 5A), thus indicating that EstDZ4 is an esterolytic enzyme.

EstDZ4 was then purified in soluble form by immobilized metal affinity chromatography (IMAC) followed by SEC (data not shown) and pure protein was used for all subsequent biochemical characterization experiments. Substrate specificity of the new esterase was evaluated by performing enzymic reactions using pNP esters of fatty acids of various lengths as substrates in the standard reaction. As shown in Figure 5B, EstDZ4 exhibits esterolytic activity against small and medium chain lengths (C2–C12), with an apparent preference for pNP-octanoate.

**Figure 6:**
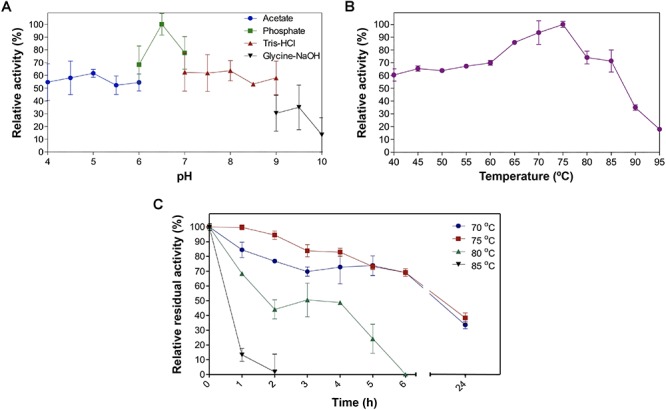
Biochemical properties of EstDZ4. **(A)** Effect of pH on EstDZ4 activity. Enzymic activity was measured in the standard reaction, at pH values ranging from 4 to 10, using the indicated buffers. **(B)** Effect of temperature on EstDZ4 activity. Enzymic activity was measured at temperatures ranging from 40 to 95°C in the standard reaction. **(C)** Thermostability of EstDZ4 was evaluated by measurements of its esterolytic activity in the standard reaction after exposure to 75, 75, 80, and 85°C for up to 24 h. The reported values correspond to the mean value from three independent experiments performed in triplicate and the error bars to one standard deviation from the mean value.

Assaying the esterolytic activity of EstDZ4 within the pH range of 4–10 at 40°C using pNP-octanoate as the substrate, revealed that the optimal pH for the new enzyme is pH 6.5 (Figure 6A). Measurements of its relative catalytic activity at different temperatures, on the other hand, showed that EstDZ4 has a broad temperature range of action as it retains high levels of esterolytic activity at temperatures between 40 and 85°C, with an optimal temperature of action at 75°C (Figure 6B). In order to evaluate the thermostability of EstDZ4, the enzyme was incubated for prolonged time periods in high temperatures and its residual activity was measured. As shown in Figure 6C, EstDZ4 exhibited a half-life of ∼5 h when exposed to 80°C, and even after 24 h of incubation at 70 and 75°C, the enzyme retained more than 40% of its initial activity, demonstrating that EstDZ4 is a highly thermostable esterase.

### Novelty of EstDZ4

The characterization of EstDZ4 as a novel enzyme does not imply the discovery of a new biological catalytic reaction or a new microbial species (although it might very well have originated from one) but derives from the fact that an enzyme that had not been described before (only 23% similar to its closest characterized entry in UniProt/Swissprot database) was isolated from a vast gene pool of numerous different microbial species originating from the same environmental sample. Bioinformatic-based predictions concerning enzymatic function are based either on the entirety of the sequence or on smaller conserved motifs (e.g., protein domains) of already known enzymes so the discovery of a totally novel enzymatic activity is a rather unachievable task if tackled solely by *in silico* approaches. However, these approaches remain the cornerstone of such an endeavor as their lists of potential enzymes provide the starting point for further curation via wet lab protocols. Such a curation aims not only to confirm each gene product’s predicted enzymatic function and to pinpoint its optimum conditions (temperature, pH, etc.) but also to search for new additional putative target substrates, potentially uncovering novel enzymatic activities. Similarly, a novel species cannot be determined with 100% certainty only by the bioinformatic paradigm, although several strategies have emerged (Droge and McHardy, 2012). In order to discover and properly characterize a new species, prior culturing is needed but this is not usually possible for most of the microbial species in an environmental niche. Nevertheless, this is exactly the type of issue that ANASTASIA’s pipelines aim to bypass. Detecting novel gene products, such as EstDZ4, from metagenomic samples whose species cannot be cultured and assigning functional properties to these sequences is one of the many different objectives of this platform. The choice of metagenomic sample to mine for enzymes of potential interest is equally important. One of the key features of EstDZ4 that makes it highly interesting is its thermostability which can be attributed to the physical properties of the site where the metagenomic material was sampled from.

### Comparison of ANASTASIA With Other Metagenomic Solutions

**Figure 7:**
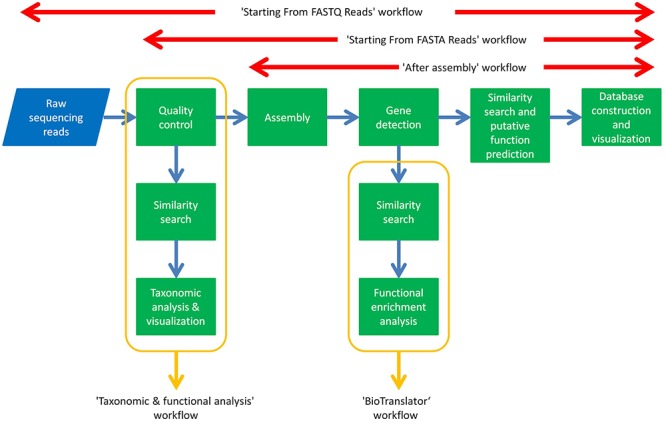
Summary of ANASTASIA’s workflows.

The idea of building automated workflows for metagenomic analysis is not a new one and there is a number of older (Ladoukakis et al., 2014) and newer solutions available (Kultima et al., 2016; Lugli et al., 2016). Most of these solutions, however, represent mostly academic compilations, requesting from their users a varying degree of familiarity with (bio)informatics programming/scripting, in order to be able to install, execute, and properly parse their resulting datasets since they operate only via Linux command line (Li, 2009; Treangen et al., 2013; Kultima et al., 2016), and some of them even comprise tools or workflows that focus only on specific parts of the metagenomic analysis (see Table 1). A very recent example is another galaxy-based platform: ASAIM (Batut et al., 2018), which includes integrated tools for every part of a complete metagenomic analysis from sequence assembly to sequence annotation but its automated workflows don’t include most of them as they mainly focus on taxonomic and functional analysis, leaving the task of novel enzyme mining up to the user’s experience in dealing with the rest of stand-alone tools. Even the Galaxy platform, which ANASTASIA has based its development on, has a limited arsenal of integrated tools for metagenomic analysis on its official public server^6^ and it requires an experienced user to download and install the platform in order to customize it accordingly for a complete analysis. On the other hand ANASTASIA offers an all-inclusive set of tools and automated workflows (Figure 7) tuned to be used to tackle each separate task of a metagenomic study, from assembling raw sequencing reads to fully annotating and predicting the function of the gene coding sequences derived from each sample. In addition our platform can be easily used even by the most inexperienced researcher, as it is available on a public server (motherbox.chemeng.ntua.gr) via an intuitive graphic user interface and offers built-in ready-to-use automated workflows with default parameters that can handle most type of datasets. This setup also includes the online data management system mentioned before, that allows the user to manually access and curate the otherwise hard-to-handle resulting datasets from an analysis. In accordance with most command-line pipelines and its Galaxy predecessor, ANASTASIA can also be locally downloaded, installed in any Linux system and even be further customized by highly proficient, bioinformatic, power users providing the same graphic user interface and data management system as on the public server. Table 1 demonstrates the strengths of the platform in comparison with the most common open-source metagenomic pipelines already available.

**Table 1:** Comparison of current bioinformatic pipelines for metagenomic data analysis with ANASTASIA.

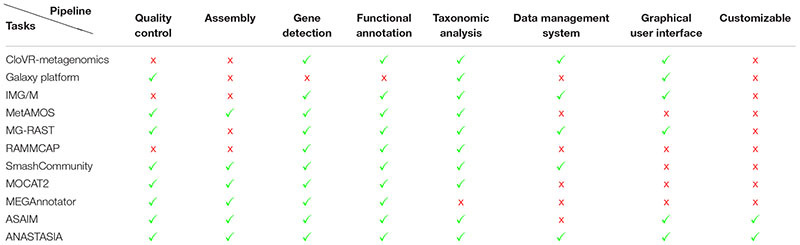

## Conclusion

Sequencing technologies have emerged to become an indispensable tool for metagenomics revolutionizing the ways, through which we can probe an environmental niche and extract more inclusive information about its genomic content for an ever decreasing cost. This evolution however, is followed by the immense increase of the generated amount of data and the need for numerous different bioinformatic tasks, which are essential for its complete annotation. Here, we present a powerful solution to these issues, the ANASTASIA platform. ANASTASIA is a portable web repository for large metagenomic datasets, providing automatic, bioinformatic workflows for data handling, and major annotation tasks via a friendly GUI. ANASTASIA functions as an intuitive and easily accessible tool, both for biologists and other users needing to store, manage and fully annotate very large metagenomic datasets, as those generated and compiled during the HotZyme project, where it was utilized for the exhaustive analysis of sequencing data from various metagenomic samplings around the world. ANASTASIA, as an automated analytical platform, represents a stable and well tested environment for the future integration of families of newer and faster algorithms, addressing diverse bioinformatic tasks emerging as pressing needs in current, ever-increasing in complexity, and metagenomic studies.

## Author Contributions

All authors listed have made a substantial, direct and intellectual contribution to the work, and approved it for publication.

## Conflict of Interest Statement

The authors declare that the research was conducted in the absence of any commercial or financial relationships that could be construed as a potential conflict of interest.

## Footnotes

1 hotzyme.com

2 https://bitbucket.org/galaxy/galaxy-dist/

3 http://toolshed.g2.bx.psu.edu/

4 http://motherbox.chemeng.ntua.gr/anastasia_dev/

5 https://bitbucket.org/TYRANISTAR/anastasia/

6 https://usegalaxy.org/
